# Multi-Aspect Based Approach to Attack Detection in IoT Clouds

**DOI:** 10.3390/s22051831

**Published:** 2022-02-25

**Authors:** Vasily Desnitsky, Andrey Chechulin, Igor Kotenko

**Affiliations:** St. Petersburg Federal Research Center of the Russian Academy of Sciences (SPC RAS), 199178 St. Petersburg, Russia; chechulin@comsec.spb.ru (A.C.); ivkote@comsec.spb.ru (I.K.)

**Keywords:** attack detection, IoT, network security, cloud

## Abstract

This article covers the issues of constructing tools for detecting network attacks targeting devices in IoT clouds. The detection is performed within the framework of cloud infrastructure, which receives data flows that are limited in size and content, and characterize the current network interaction of the analyzed IoT devices. The detection is based on the construction of training models and uses machine learning methods, such as AdaBoostClassifier, RandomForestClassifier, MultinomialNB, etc. The proposed combined multi-aspect approach to attack detection relies on session-based spaces, host-based spaces, and other spaces of features extracted from incoming traffic. An attack-specific ensemble of various machine learning methods is applied to improve the detection quality indicators. The performed experiments have confirmed the correctness of the constructed models and their effectiveness, expressed in terms of the precision, recall, and f1-measure indicators for each analyzed type of attack, using a series of existing samples of benign and attacking traffic.

## 1. Introduction

Currently, the integrated systems of the Internet of Things are becoming more and more widespread, including many remotely located networks of heterogeneous devices operating in an untrusted environment. Such devices interact locally and perform communication and business functions by using primary and auxiliary cloud services. For example, a typical Smart Home system includes, firstly, lots of microcontrollers with installed sensors and actuators for collection, processing, and control of the premises infrastructure at the physical level; and secondly, various means of local and remote access to data and network functions implemented as web services, software applications, hardware GUI elements, etc.; and, thirdly, gateway equipment for interacting with the cloud and providing remote access to the network from the outside. A list of the full forms for the abbreviations use in the article is given in Abbreviations section.

Typically in IoT networks, there are multiple access points that can be exploited by potential attackers to influence the devices. Such access points can be located both within the communication perimeter of the network, and remotely—at a short distance by using wireless communication protocols, or via the Internet and cloud infrastructure. This circumstance makes such networks especially vulnerable to various types of malicious influences. We also note that, unlike general-purpose local area networks, modern IoT infrastructures, as a rule, are characterized by the complexity of embedding protection means into their devices. This means they assume significant constraints on their resources and are limited in software compatibility. As an essential factor of IoT clouds, one can single out the dynamic nature of their functioning, including the ability to change their composition, location, device settings, data, profiles, features of user behavior, and the structure of data flows. In particular, this is due to both the technological and the organizational complexity of updating such devices, which often involves manual servicing of the device and updating the firmware to eliminate vulnerabilities. All this necessitates the development of specialized approaches to ensuring information security of IoT clouds, which would take into account their features and allowing not only the prevention of possible attacks but also effectively detecting and reacting to the threat [1,2]. At the same time, such detection would make it possible to warn and make decisions about the security of end devices, both by the operator/providers of the entire IoT infrastructure and by end-users.

Therefore, in addition to the need to support the automation and intelligence of an IoT network, information security functions are also imposed on the cloud infrastructure. The main advantages of using the cloud and its services to ensure the security of IoT clouds include, first, the virtually unlimited computing resources of the cloud, which provide the need to deploy computationally intensive protection algorithms, and, second, the ability to use the cloud for collection and group-based analysis of data from many similar or different IoT clouds. In addition to analyzing Big Data and identifying various patterns that affect the servicing of IoT clouds and the needs of users, the cloud infrastructure also allows the detection of distributed coordinated attacks. Such attacks may appear in the form of botnets and complex multi-step attacks, in which tens of thousands of devices of different IoT clouds can be involved, involving SYN port scanning, SYN portsweeping, and SYN flooding. It seems that it would be extremely difficult or almost impossible to detect such attacks locally within the equipment of each specific IoT network individually.

At the same time, such cloud services for information security have certain disadvantages. In particular, these are the inherent communication limitations of network communication channels, the practical impossibility of providing the entire completeness of network security data sent to the cloud. It causes the need to extract, aggregate, and send only some minimal necessary data to the cloud. In addition, privacy issues impose additional legal and marketing restrictions on the ability to send any user data to the cloud or even small fragments of such data. In particular, even the fact of sending completely anonymized user data may cause both justified legal questions to the provider and negative reputational costs due to not enough end-user awareness.

Therefore, a possible way to organize the protection of an IoT network is considered to be the decentralization of information security and attack detection means. In particular, it allows some of the protection modules to be placed directly on user devices, some other parts could be located on the central device of the IoT network/routers/other network equipment, while the rest would be directly in the cloud. This, in turn, creates the following technological challenge: how should the functions of information security as well as network data collecting, processing, sending, and storing procedures with their inherent limitations, be distributed among the three specified groups of entities [3]? This leads to another technological challenge we aim to solve in this article. Within the framework of conditions and severe restrictions on data and available resources commonly being set by the developers of the entire IoT ecosystem and its providers, how to effectively detect attacks and abnormal device activities in the cloud [4]? The work in the cloud has certain advantages and disadvantages, namely a lot of computing resources and a lack of detailed information about traffic because of privacy issues. In addition, an important and urgent task associated with this technological challenge is to obtain the most detailed information about attacks, including the type of attack, attack starting time, etc.

The main contribution of this article includes a proposed approach to multi-aspect attack detection based on the construction and combination of heterogeneous training models. Each model is formed by analyzing the original traffic, extracting and constructing a specific space of features of various attacks. The novelty of this approach includes (i) types of the selected network attacks being an integral part of complex combined multi-step actions; (ii) extracted and constructed sets of features for these attacks, reasoning from the characteristics of TCP/IP traffic of IoT clouds; (iii) combination of particular adapted common classification methods based on machine learning. In addition, unlike existing analogs, the simultaneous analysis of various aspects of TCP/IP traffic in IoT clouds allows not only detecting an attack but also determining its main characteristics. At the same time, the novelty of the approach is expressed in the improvement of the detection quality indicators, which is confirmed experimentally.

Also, the advantage of the proposed approach lies in the fact that it can take some statistical information about the session as an input instead of the full traffic, which includes the payload. This feature has two sides. On the one side, this approach allows one to resolve partially privacy issues and reduce the amount of transmitted data. On the other side, it can reduce the quality of attack detection.

The rest of the article is organized as follows. Section 2 presents the statement of the problem, requirements, targets, and features used in the process of attack detection. Section 3 discloses an overview of the relevant literature in the field. Section 4 describes the proposed approach and training models. Section 5 describes software implementation. Section 6 discloses experiments. Section 7 presents the outcome of the analysis of the results obtained in this work. Section 8 concludes the article.

## 2. Task Statement

### 2.1. Requirements and Target Indicators

The work aims to build a set of training models and a framework for detecting and automatic analysis of network attacks in IoT. A typical infrastructure represents a TCP/IP network and includes devices that can be either the object of an attack or used by an attacker to perform actions against other devices. The infrastructure includes a central control device usually represented by a router and is responsible for performing core network management functions. In particular, the central control device is in charge of (i) communication of the IoT network with the cloud, providing the centralized control and application services, (ii) routing/physical passage of data flows between IP network devices and flows targeted to or from the cloud, (iii) user access to the network settings.

The training models are built based on known traffic datasets by using various artificial intelligence detection methods [5,6]. To build the training models the input data with sets of TCP/IP traffic features are marked as data samples of benign and attacking traffic, respectively. These features form various aspects (i.e., specific spaces of various attack features) and should be used in the process of training and testing the models. At the output of the learning process, a specific software module is obtained allowing the detection and determination of given types of attack with certain values of the detection quality. Therefore, models correctly trained and tested in experimental studies can be used as a part of attack detection tools in real IoT clouds. The input data of such a detection tool are flows of the TCP/IP traffic feature collected by the central device and received dynamically during the operation. The output data includes flows of events and warnings about the presence of an established type of attack or potentially suspicious actions in networks. This data is then logged onto the cloud.

The supposed location of attack detection tools within the cloud-based structure allows for not only optimizing the performance and resource consumption of the detection processes and getting an ability to effectively balance the computational load but also to detect coordinated attacks performed simultaneously by using several or many geographically located remote IoT clouds connected to this cloud.

One of the most important requirements imposed on an IoT network device to detect network attacks in its traffic is the need for the device to be involved in the communication with both a router and remote entities in the cloud. In addition, to be able to monitor the state of a device in the cloud, it is also necessary that the business logic of this device depends not only on the current readings of the sensors installed on it but also on network requests to it.

Moreover, it is assumed that the ecosystem of IoT network devices includes not only TCP/IP devices but also some simpler software/hardware modules that make communications outside via TCP/IP devices by using Bluetooth, ZigBee, and other protocols. As a result, the traffic from such units is mixed with the traffic of the devices that these interact through. Moreover, this circumstance should be taken into consideration when building the training models.

The requirements also include the need to form a combined training model that allows for multiclass traffic classification based on a variety of possible attacks. The sufficiency of the volume and the correctness of the structure of the training traffic should be checked through experiments. In addition, the traffic samples used should be sufficiently representative of the inference function of the classifiers. It would allow correct recognition of attacks in the traffic of various types of IoT devices at the exploitation stage. Particularly attacks should be identified also in the traffic generated by devices after legitimate firmware and software updates of the device without the need for reconfiguration and adaptation of the detection mechanism [7].

### 2.2. Attack Features

Since attacks should be detected centrally, i.e., in the cloud, and the resulting practical impossibility of redirecting a copy of all traffic passing through the router to the cloud, the router can generate only some limited portions of data. This data characterizes the current traffic and is sent to the cloud as soon as network sessions appear and terminate. In addition to limitations on the amount of information transmitted outside the IoT network, the restriction on the privacy of both service-related and user data of device traffic is extremely significant. As a result, the prohibition imposed by the IoT service provider on any potential disclosure of information—including domain identifiers, POST/GET parameters, and other important characteristics such as packets headers and payloads –significantly limits the structure and content of the input for the attack detector. Note that this circumstance can lead to a decrease in the values of the detection quality indicators for all or a part of the detected attacks. It could even make it impossible to detect some of them.

In general, data sent to the cloud is formed in accordance with the existing typical network traffic accounting protocols, such as NetFlow [8] or NetStream [9], possibly with some restrictions. In fact, generated data flows include fields (i.e., traffic features) that identify incoming and outgoing network sessions, as well as many statistical characteristics of packets. Note that the possible limitations imposed by the provider to optimize performance indicators include the ability to send features only generated and calculated for the first minute of each session to the cloud. Table 1 exposes features that were selected based on the analysis of the initial data of an existing cloud-based service for the ecosystem of Home Network IoT devices.

### 2.3. Attacks

Based on the typical list of traffic features sent to the cloud side and given in Table 1, the following list exposes the main types of attack that are possible and reasonable to detect based on them:

(1) Basic network and transport layer attacks, including scanning and flooding attacks.
(2) Some particular types of application-layer attacks, such as Brute Force attacks on SSH, HTTP, etc.
(3) Some multi-step network and vulnerability exploitation attacks.
(4) Coordinated (cohered) attacks involving the operation of a certain type of device from various IoT clouds.

Note that, given the practical complexity, laboriousness, and the lack of guarantee of the validity of the initial data for a large number of attacks, for the formation, assessment, and improvement of training models it seems appropriate to focus on the existing records of attacking traffic available on the Internet, even if recorded for different types of IoT systems, rather than to simulate each attack on available resources. Nevertheless, to use existing traffic records, it is necessary to adapt them, including the modification of individual traffic fields, mixing several types of traffic, its replication at a certain time interval, and other types of transformations. The particular attacks we used in the experiment are the following. These are SYN port scanning attack, SYN portsweep attack, SYN flood attack. Within an SYN port scanning, the attacker performs scanning by SYN packets with no termination of the connection. Such scanning appears to be quite effective and is not detected by the most simple event detection means. A portsweep attack may be applied to scan a concrete port or several ports on a huge range of devices for instance to disclose any specific application services running on them, possibly as a starting action of a more sophisticated multi-step influence. Analogously to the former one, the essence of the SYN flood attack is to send multiple SYN requests to the victim device with no reaction on SYN + ACK responses, therefore overflowing the victim’s connection buffers, blocking it from the normal network communication.

## 3. State-of-the-Art

At present, the detection of attackers in modern networks is mostly reduced, first, to the analysis of network traffic of devices, in most cases using TCP/IP traffic, and, second, to the use of host-based detection tools [1,2]. Nowadays, intrusion detection systems are actively used. Such systems are based primarily on a set of rules and allow the identification of specific types of illegal influences on the basis of specified signatures [10]. The disadvantage of such an approach is its low adaptability, i.e., when a small modification of the attacker’s actions, conditions, and parameters of the attack, the attack is likely not to be detected.

At the same time, there is an increasing trend towards research and attempts to put various intelligent detection methods into practice [5,6,11]. The advantages of such methods include their focus on detecting not only a given, fixed list of attacks based on existing rules and signatures, but also a part of previously unknown attacks that may exploit zero-day vulnerabilities. However, when using these intelligent methods, the probability of errors due to false positives and false negatives increases. False positives are also often considered especially critical [12].

The lack of development and applicability of these methods, in comparison with ones that are simpler and widely used in practice, are associated with the previously unpredictable result of their work. Furthermore, typically each tuned specific intelligent method is focused only on certain specific types of attack. In addition, the complexity of building effective intelligent methods for detecting attacks is associated with the need to check and improve the detection quality indicators by refining and constructing new specific attack attributes, refining the hyper-parameters of the algorithms used, and improving the balance, volume, and quality of the initial data. In particular, to overcome the lack of adequate initial data, various sampling techniques are used. These techniques make it possible to additionally balance the distribution of data between different classes in the training set. Note, the presence of a dataset of sufficient size and its correctness provide the necessary inference ability of classifiers used to detect attacks.

Often, attack detection is performed on statistical and historical data from network devices. In conditions of the absence of such historical data, an approach to detecting attacks based on reinforcement learning is also used [12]. In addition, even in a case of a sufficient amount of historical data, they can become outdated fairly quickly, thus it may lead to a lack of the initial data. According to the reinforcement learning approach, the most relevant (fresh) data and/or their labels are received immediately at the time of training, as a result of a response of the training module requesting the current data from the devices.

If it is impossible to obtain sufficient initial data, an approach to detecting attacks based on transfer learning is also used [13,14]. According to this approach, the training is fulfilled on data got from some similar object or model. After that, additional experiments are performed to check and adjust the constructed classifier for the target object.

In the absence of data labeling, which is usually used for supervised learning, methods of clustering and one-class learning are used. In particular, some of such methods are known as an apparatus for detecting anomalies, i.e., suspicious data deviations. Additionally, these anomalies could be also regarded as possible features for attack detection [11]. A significant peculiarity of such methods is that they allow one to rely not on previous (i.e., labeling for the supervised learning) or current experience (as responses from the environment—in the case of the reinforcement learning), but on identifying structural features and internal relationships in data without explicitly assigning their samples to one or another class.

Note that such approaches can be used to detect attacks not only in conditions of existing limitations on the initial data but also in order to obtain additional detection methods based on alternative principles. This implies the need to combine the existing heterogeneous detection methods. This combining is aimed at improving the detection quality indicators. The methods of combination can include both straightforward methods of majority/weighted voting and various sophisticated ensemble methods, which use specialized machine learning techniques such as bagging, boosting, AdaBoost, blending, stacking, etc. [15,16].

To solve the attack detection problems, the use of various detection methods, including combined ones, requires mandatory experimental studies on some new, independent sets of initial data. This is important since even the use of techniques that are considered as means of improving the quality of the detection (as, for example, in case of imbalance in the initial data), can have negative effects, such as the effect of overfitting, which worsens the results.

As a result, in the absence of improvement of the detection quality indicators, one can make corrections of (i) the initial data, the principles by which they are formed or preprocessed by, samples or volumes of this data, (ii) the used features of attacks and the device normal behavior, (iii) the detection methods and their hyper-parameters, and (iv) the methods of their combining. At the same time, at each of these four stages, one should take into consideration the specificity of a given class of IoT devices, i.e., sources of the analyzed data, modes of their operation and usage, as well as the specificity of detected attacks.

Note, the existing combined methods for detecting attacks in the IoT can show fairly good outcomes for certain types of attack, but not for all of them. Therefore, to improve the quality of detection, it seems promising not only to expand the existing feature space by adding new, previously unused data, or constructing derived features based on statistical processing of existing ones but also to transform the set of analyzed objects itself. For instance, it can be a transition from traditionally analyzed network sessions to hosts, networks, data flows, packets, users, types/models of IoT devices, etc. [1,17,18,19].

In [20] the following four main groups of methods that can be used to detect attacks and anomalies in network traffic are singled out: (i) behavioral methods that use the profiles of benign activity by networks, devices, and users, taking into account significant deviations from them in the detection process; (ii) methods based on knowledge, rules, and signatures; (iii) machine learning-based methods, including supervised learning, clustering, regression methods; (iv) methods of computational intelligence based on genetic algorithms, fuzzy logic, immune systems.

In [21], a dynamic multi-class classifier model is proposed for detecting several types of Network Intrusions, on the basis of a combination of basic binary classifiers built on seven well-known machine learning algorithms. The mechanism of selection of the most preferred models (i.e., individual ML-based classifier) is used depending on the values of the classification quality indicators for each considered type of intrusion. At the testing stage, 7 pre-trained models and combined models run in parallel, and the best classifier is selected. The classifiers are combined on the basis of voting and ensemble ML using the XGBoost method. This ensembling showed the best results and was therefore chosen as the basis for the dynamic classifier. Specifically, the TPR indicator exceeded 0.9 for DoS attacks, scanning attacks, network worms, and some types of exploitation of access control vulnerabilities.

The dataset UNSW-NB15 [22] was used as the initial data in [21]. It includes benign and attack traffic of several types [23,24]. The following types of features are taken as a base: basic features, time features, flow features, content features, additional features, and labeled features [25]. In order to ensure the performance of the training and classification algorithms, the authors selected only the most important features. In particular, the authors used features built per session on the basis of statistics of some fields of TCP/IP headers and application layer headers. These include session duration values, protocol, and application service numbers, session state, number of packets in a session, average packet length, TTL, and others. The categorical values of the fields are reduced to numerical values, and the numerical ones are transformed to z-score values calculated as the number of standard deviations from the mean value of the feature.

In [26], to solve the problems of detecting attacks and anomalies in traffic, a two-stage architecture is proposed. The architecture uses benign activity profiles and methods of one-class classification. This architecture is designed to achieve a tradeoff between the quality of detection and the computational resources spent on it. During the first stage, it uses a faster classifier, which ensures the revelation of known and unknown attacks with a high detection recall value, but with a possibly large number of false positives. After a possible attack is detected, a refinement is performed by using a second, more resource-intensive classifier to reduce the number of false positives. The raw data is network connections derived from existing known datasets (KDD’99 and NSL KDD). It includes basic header fields, payload elements, connection timestamps, and data on hosts. The authors use, first, metric-based methods of one-class classification based on the calculation of proximity metrics, and, second, variations of one-class support vector methods, where the experiments demonstrated rather high values of the precision indicator and various values of false-positive rate.

In [27] Kanev, et al. explore anomaly and attack detection mechanisms in wireless Smart Home networks. Among the considered features of network traffic, the authors note the following as the most important ones, namely (i) a number of data packets received and sent by the device per unit of time; (ii) a number of lost and erroneous packets per unit of time; (iii) values of the power of the outgoing signal and power consumption of the device per unit of time. As part of experiments on data obtained by simulating an attack on a sensor in the OMNET++ environment, the use of K-nearest neighbors, Random Forest, and neural networks allowed the authors to build an algorithm for detecting the simulated attack, getting an indicator of the area under ROC curve equal to 0.9689.

In [28], the complexity and potential inefficiency of deploying intrusion detection and malware detection tools by using signature-based methods in IoT systems are substantiated. The complexity of the implementation of such tools is due to both the limited hardware resources of IoT devices and a significant increase in energy consumption as a result of the use of cryptographic security tools. At the same time, methods for detecting intrusions and various types of attack on IoT infrastructures using machine learning methods seem to be more and more promising [29,30]. At the same time, in order to address specific types of attack, simulation and natural modeling is used, with which it is possible to generate data sets necessary both for constructing training models and for testing their effectiveness [31].

It is shown in [32] that the use of clouds for intrusion detection in IoT networks allows for the improvement of the quality of detection, compared to traditional methods which use the cloud as an environment for centralized processing and analysis of the entire completeness of available data [33]. At the same time, the presence of restrictions on the privacy of data, and the resulting impossibility of achieving the required completeness of the source data in the cloud, as well as the technical complexity of transferring long traffic records and logs to the cloud due to the limitations of the bandwidth of communication channels and performance [34] of devices complicates the effective cloud detection of attacks for many IoT networks. Note that, in contrast to existing works, in this article we are trying to achieve a certain balance between the quality of detection, on the one side, and on the other, the availability and privacy of the initial data by fine-tuning the ensembles of the classifiers we have formed.

The key peculiarity of this article that distinguishes it from earlier efforts is the development of a multi-aspect approach to the detection of IoT attacks on the basis of introduced aspects and their combination with the use of intelligent data analysis methods. On the example of the attacks analyzed in this work, it is experimentally proved that the proposed approach is superior to any single-aspect classification in terms of detection quality. Moreover, it appeared to surpass any single-aspect combination of various detection methods and their hyper-parameters.

Additionally, a distinctive feature of this article is the limited initial data used to obtain the attack features to analyze. Due to privacy restrictions, during the detection process, only a part of the features collected according to NetFlow and NetStream specifications. Therefore, in the process of their construction, the training models for attack detection were adapted not only for specific types of attack but also under strict constraints on the initially available data.

Finally, the specificity of the article also represents the combined nature of the proposed attack detection mechanism. It is expressed firstly, in the implemented multiclass classification for a number of attack types, and secondly, in the combined use of statistical methods for detecting attacks and ones based on machine learning. The performed experiments have shown that the constructed multi-aspect classifiers ensure the achievement of acceptable and high indicators of detection quality.

## 4. Approach and Models

**Figure 1:**
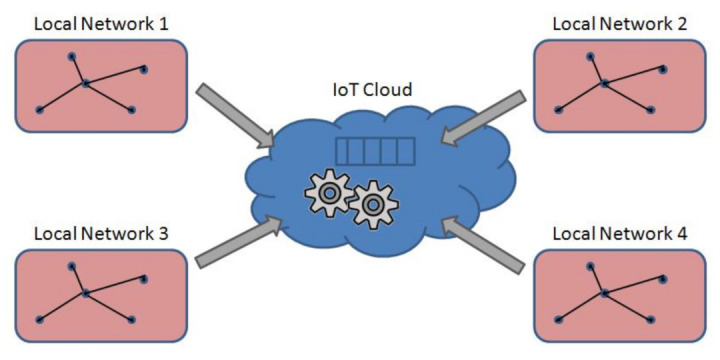
System model.

This section proposes an approach to attack detection based on the ensembles of machine learning methods applied in parallel to various aspects of network traffic. The underlying system model exposing the network infrastructure for the approach is illustrated in Figure 1. The model presents a range of local networks with IoT devices and the cloud to collect, process, and analyze data on IoT traffic features coming from the local network side.

**Figure 2:**
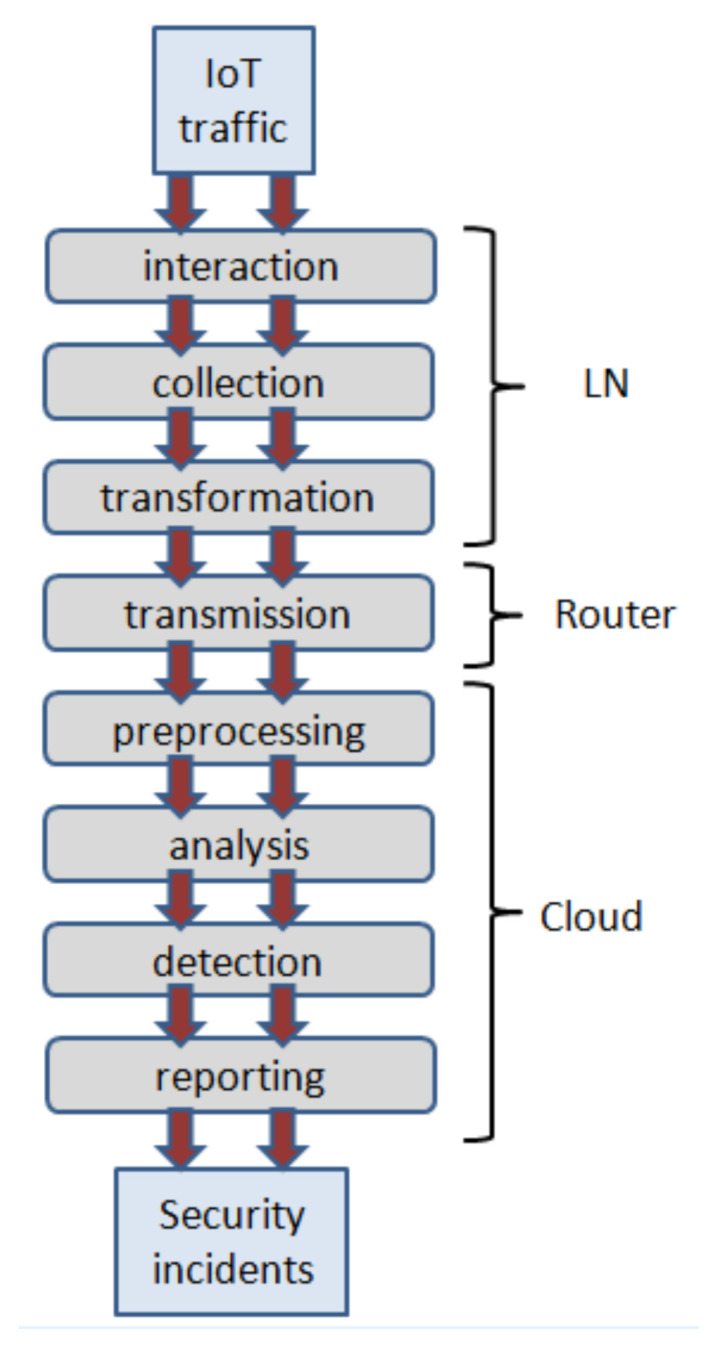
Functional system model.

Figure 2 illustrates a functional diagram describing the main targeted processes performed in the model. IoT traffic features being a source data are passed and transformed within the target processes sequentially, starting from a phase of device interaction, data collection, and transformation performed in local networks connected to the cloud. Further, it is subjected to data transmission through the router bordering other local network devices and the Internet. After passing to the cloud side the data from numerous local networks are preprocessed and analyzed in order to perform the detection. Finally, the detection results are reported to an administrative entity in a form of specific security incidents disclosing the particular fact of the attack and related information. Besides the whole sequence of these processes runs in parallel dynamically for data formed and received at different time points. Note, data accumulation and aggregation for their further group-based procession are done on the analysis, detection, and reported phases.

**Figure 3:**
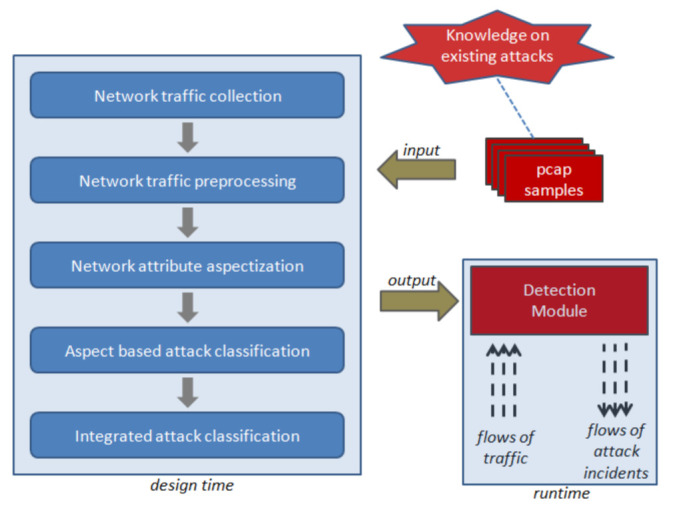
Scheme of the attack detection approach.

An ensemble is understood as a classical machine learning a combination of classification algorithms in order to obtain the best quality solution to the attack detection problem. The approach involves the implementation of the following sequentially performed stages. Each stage is focused on performing its own subtask and is schematically shown in Figure 3. The diagram presents the generalized stages of the formation and configuration of the proposed comprehensive approach to attack detection. The input data represents samples of tagged TCP/IP traffic containing records made as a result of normal device operation and in case of attacks. The output data is a trained and tested classifier capable of detecting the considered types of attack with the specified precision indicators and detection time.

At the exploitation stage, the resulting detection component represents a single module deployed on a network host and converting information about the current traffic from network devices into specific security incidents by means of the trained integral classifier. Each incident includes the name and type of the attack, timestamp, and other related information about its occurrence. Let us describe the following main stages.

### 4.1. Stage 1. Network Traffic Collection

At this stage, network traffic is collected by using specially configured network equipment. The data collection is usually performed on one or more devices of the local IoT network, such as a network router, through which all communication between network devices and the existing cloud network infrastructure is fulfilled. It should be noted that at this stage, the completeness of the collected data is limited by the physical passage of network streams through the data collection point and the possibilities of using traffic redirection/mirroring techniques.

### 4.2. Stage 2. Network Traffic Preprocessing

At this stage, the main features of network traffic are identified. For this, specific network traffic preprocessing procedures are performed. They transform its attributes into a set of high-level features, such as the intensity of network exchange between hosts, port scan statistics, combinations of flags set in packets, errors in establishing TCP connections, etc. A detailed list of features is given in Table 1.

Note that at this stage, the completeness of the collected data is limited primarily by the peculiarities of the used versions of the NetFlow/NetStream protocols that provide such collection, and by the settings of the installed modules of the network sensors and collectors of the gathered data.

### 4.3. Stage 3. Network Attributes Aspectization

An analysis of existing studies, together with a series of preliminary experiments conducted by us to detect various types of attack directly based on the features in Table 1 with the use of intelligent data analysis methods, allow us to conclude that the quality of such detection remains insufficiently high for most types of attacks. In addition, even the presence of a sufficient volume of internally balanced labeled datasets, even in case of the correct detection of an attack incident, does not allow determining the type of such an attack with high accuracy. This is due to significant differences in the sets of features on which the detection of various attacks should be based. As a result, it becomes necessary to introduce a concept of an aspect covering attack detection based on sets of direct and derived attack features.

In addition, operating exclusively in terms of a session, for detecting a number of attacks, in addition to clarifying the fact of an attack, it is difficult to determine its source hosts and distributors, as well as other related information that may be needed in the future to react to the attack.

Thus, since a different set of features can be effective for detecting different types of attack, it is necessary to form their groups; an action that is performed at this stage. For example, for detecting a distributed denial of service (DDoS) attack, incoming traffic is the most important, for scanning detection—it would be outgoing traffic, and for detecting the use of exploits—the most considerable are individual sessions of network traffic. Thus, each aspect represents a set of features that reflect some view on the object to be checked and contains all the information on the analyzed network activities. For example, these can be features that characterize the intensity of network connections from multiple hosts to one host within a DDoS attack. Network traffic, presented as a set of aspects, is ready to be processed directly by machine learning.

Table 2 provides an example of a specific feature space based on the features from Table 1 to detect Distributed Denial of Service (DDoS) attacks. Note that since Table 1 lists the features characteristic of a separate TCP session, in this aspect of network traffic a separate record is the statistics of network connections for a time of T seconds with a step of S seconds for one dstIP within the protected network.

This aspect allows for detecting the presence (but not an exact list) of TCP sessions typical for DDoS attacks aimed at the dstIP address of the network for a time period of T seconds, starting from startTime.

### 4.4. Stage 4. Aspect-Based Attack Classification

At this stage, machine learning methods are performed. It uses ensembles of classification methods designed to detect a predetermined set of attacks. Each attack type assumes its own ensemble holding one or more aspects of the network traffic. Thus, the number of ensembles of classification methods for attack detection is equal to the number of attacks, and each ensemble takes a specific subset of aspects as an input. Each ensemble of classification methods is a set of basic classifiers and defines a meta-algorithm consisting of all the individual machine learning methods used. The way they are used together can be different (voting, boosting, stacking, etc.) and are chosen to ensure the efficiency of the final solution.

The stage is divided into 2 phases, namely training and running. At the first (preparatory) phase, each of the ensembles is trained on a specific attack—by using a required set of aspects. In the second (main) stage, attacks are directly detected based on aspects of network traffic obtained in the previous stage.

As a result of the work, each of the ensembles forms its own probabilistic predictions regarding the fact that one of the specified attacks is in the traffic.

### 4.5. Stage 5. Integrated Attack Classification

Since each of the ensemble groups will work best when only the attack that the group is targeting appears in the traffic, the detection efficiency will be reduced in a real network with many attacks. For example, in some cases, attacks can have overlapping time ranges (for example, they can occur simultaneously). In other cases, due to common features, some attacks will introduce noise into data, which classifiers of another attack operate on, etc.

To prevent such a decrease in performance, the approach uses an integral classification, which uses at the input not the features of the network traffic, but the results of the work of ensembles of attack detection methods. For this, at this stage, an additional ensemble of classifiers is used, that is similar to the one used in stage 4. This extra ensemble taking a separate vector of probabilities for each of the attacks as input, outputs a single vector of probabilities of finding each specified attack in the network traffic.

### 4.6. Stage 6. Single-Step Attack Detection

Based on the probabilities of attacks obtained at stage 5, and using a pre-established detection condition (for example, as such condition, one can set an admissible threshold for the highest probability of one of the attacks), we can assert that there is a specific attack in the traffic. At the end of the stage, if an attack is detected, a security incident is generated.

Thus, detection of attacks in network traffic is ensured by using ensembles of basic classifiers specialized for each of the specified attacks (aspect classification), as well as an individual ensemble for correcting the results obtained by aspect classifiers of attacks (integral classification). It should be noted that the general detection architecture makes it possible to add support for new types of attack without significant modifications. It can be done by expanding the set of feature aspects (stage 3), as well as adding a new ensemble of classification methods trained on a new attack to the attack detection group during aspect classification (stage 4).

## 5. Testbed Implementation

In our experiments, the attack detection process was divided into the following four sub-stages:

1. Feature extraction and selection, i.e., the selection of only the necessary set of features from the traffic (the list of features shown in Table 1 was used in the experiment; the main steps are presented in the pseudocode below, see Algorithm 1):

**Algorithm 1.** Feature extraction and selection algorithm.**Input:** traffic dump**Output:** array of sessions with selected features Procedure FeaturesExtraction(Traffic tdump)

  sessions[]: = ExtractSessions(tdump)  dataset[][]: = [][]  i: = 0  for session in sessions   dataset[i]: = ExtractFeatures(session)   i++
**end for**

**end procedure**


It should be noticed that in the final implementation, step 1 will be performed in the hosts and all other steps will be done in the cloud.

2. Feature construction, i.e., the transformation of the data extracted from the traffic into the form necessary for the work of classifiers operating with various traffic aspects (in the framework of the experiment, the list of features given in Table 2 was used, the main steps are presented below, see Algorithm 2):

**Algorithm 2.** Feature construction algorithm.**Input:** array of sessions with selected features, time interval, time shift**Output:** extended arrays of sessions with selected and constructed features

 Procedure FeaturesConstruction(Dataset dataset, int timeInterval, int timeShift)  for session in dataset   UpdateSourceHostStatistics(session, timeInterval, timShift)   UpdateDestionationHostStatistics(session, timeInterval, timShift)   UpdateSessionsStatistics(session, timeInterval, timShift)  endfor  datasets[][][]: = [][][]  datasets[srchost]: = getSourceHostStatistics()  datasets[dsthost]: = getDestinationHostStatistics()  datasets[session]: = getSessionsStatistics()  datasets[rawsession]: = dataset
**end procedure**

3. Detection and analysis of attacks, i.e., straightforward application of multi-aspect classifiers on the prepared data to detect attacks. This complex detection mechanism includes basic classifiers, aspect-based classifiers and integral classifiers. Both separate classifiers constructed on the base of a single aspect and an integral classifier, which joins the results of the other basic classifiers, are used as follows below, see Algorithm 3:

**Algorithm 3.** Attack detection and analysis algorithm.**Input:** extended arrays of sessions with selected and constructed features**Output:** extended arrays of sessions with selected and constructed features as well as the recognized attack labels

Procedure attackDetection(Datasets datasets)  trainingSet, testingSet = datasetSeparation(datasets, 80)  result1: = DetectAttacksInTimePeriodsForSourceHosts(trainingSet, testingSet)  result2: = DetectAttacksInTimePeriodsForDestinationHosts(trainingSet, testingSet)  result3: = DetectAttacksInTimePeriodsForSessions(trainingSet, testingSet.values)  result4: = DetectAttacksInSessions(trainingSet, testingSet)  result: = ResultsIntegration(result1, result2, result3, result4)
**end procedure**

4. Formation of results, i.e., the output of detection results in the required form (the main steps are presented below, see Algorithm 4):

**Algorithm 4.** Result formation algorithm.**Input:** the training set and the testing set that contain extended arrays of sessions with selected and constructed features as well as the recognized attack labels**Output:** report

Procedure reportGeneration(DetectionResult result, Datasets trainingSet, Datasets testingSet)  resultsEvaluation: = resultEvaluation(results, testingSet.labels)  report: = reportGenerator(resultsEvaluation, results, testingSet.labels)
**end procedure**


For the experiment, we developed an experimental testbed designed to train aspect classifiers and an integral classifier to detect network attacks. This testbed implements the approach to attack detection proposed in this study.

The developed experimental testbed implements several functions, namely: (1) extracting data from a file in PCAP format; (2) preparation of additional data, i.e., forming various aspects of traffic; (3) formation of training and test samples that contain aspects corresponding to each type of attack; (4) training ensembles of classifiers for each aspect of the traffic as well as an integral classifier to form the final decision based on the training sample; (5) evaluation of the quality of trained classifiers based on test samples.

It should be noticed that in the final implementation, step 1 will be performed within the hosts and all other steps will be done in the cloud. This approach allows one to reduce the amount of the transmitted information as well as to solve the personal data issues. Additionally, the approach allows one to apply the anonymization methods without a strong impact on the detection results. However, this also leads to difficulties in the attack detection—it should be noticed that in general, scanning detection should be based on host statistics instead of session statistics. Each session (even to the closed port) cannot be recognized as an attack. However, some amount of sessions (or single packets) targeted at different ports can be recognized as an attack. So the experiment results that are presented in Table 3 were only aimed to show and to test the ability of the developed frameworks to detect attacks by the basic features and classifiers.

The main libraries required for the functioning of the experimental testbed are the following—Scikit-Learn [35] for machine learning and Pandas [36] for data processing and analysis. A file in PCAP format and two configuration files are to be passed to the testbed. The first configuration file contains the traffic parameters in the source file, while the second one contains parameters for the experiment. As a result of the work, the experimental testbed forms an array of strings that contains the mark of the detected attack and the corresponding number of the network connection.

## 6. Experiments

The aim of the performed experiments was to check the effectiveness of the approach proposed above. It should be noted that the essence of the proposed approach lies in the consistent application of ML methods to different feature spaces. The proposed approach also uses the technique based on the classifiers combination, but this combination uses standard means of combining (although the combination itself allows one to improve the attack detection quality, but it is out of scope for this investigation).

The first step in conducting the experiments was to choose a dataset with the network attack and a machine learning method. For the experiment, a portsweep attack was chosen (i.e., scanning multiple IP addresses to find one specific open port). The files containing session attributes with port scan attack content were used. The source traffic was taken from the DARPA database. The following file was used: 1_portsweep_warezclient.csv (total—26831 records; benign—24401; attack—2430).

In accordance with the results obtained, the RandomForestClassifier method was chosen for further experiments with the following parameters (see Table 4).

It is also should be noted that the investigation was focused on the features construction and the architecture of the classifiers combination. It is assumed that the usage of different ML-based classifiers like CNN or LSTM can enhance the general quality, but does not affect the general ratio of results showing the effectiveness of the proposed approach for the formation of features and the combined classifier.

The second step in conducting the experiments was to apply the chosen machine learning method to detect several types of attacks in network traffic at once. For this, training and test samples were formed, which include the following attacks: SYN port scanning, SYN portsweep, SYN flood.

**Figure 4:**
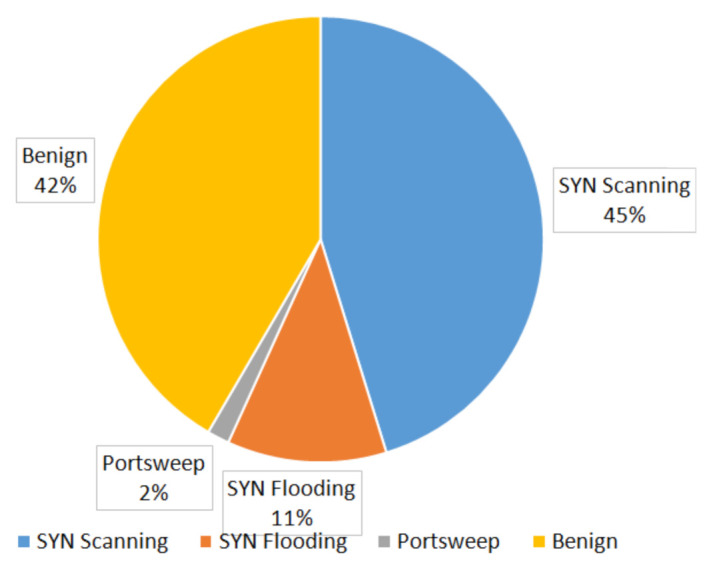
Distribution of the attacks and benign sessions in the dataset.

The joint dataset has the following distribution of the attacks and benign sessions (see Figure 4).

The experimental results showed that despite the sufficiently high quality of detection of the very fact of an attack (the accuracy is more than 0.998), the list of features from Table 1 and the approach itself based on the analysis of individual sessions do not allow for achieving a high quality of recognition for specific type of attacks (the accuracy of the multi-classification is about 0.43).

Table 5 shows the analysis of the correlation between features of the sessions and the session’s label. The main used features are the flags, connection status and source data length. It shows the logic of the classifier—if a connection has an abnormal end or it has only an ACK flag it means that it is an attack. However, these features cannot help to distinguish SYN flood and SYN scanning attacks.

The third step of the experiments was to apply the proposed classification scheme, which includes multi-aspect input data, and individual classifiers for each type of attack in the network traffic. For this, training and test samples were formed, including the following attacks SYN port scanning, SYN portsweep, SYN flood.

The results of the experiment showed that although aspect classification does not allow detecting a separate attacking session, it shows a sufficiently high quality of attack detection (the accuracy of the multi-classification is more than 0.9999 for the dataset generated in the testbed). It is due to the recognition of a specific type of attack. However, the dataset was grouped by the time intervals and these intervals were marked as scanning if they have at least 10% of scanning sessions. This approach can show the presence of the attack, but cannot be used for the session blocking because of false positives from the benign sessions in time intervals with scanning sessions.

The fourth step of the experiments was to apply the proposed classification scheme, which includes multi-aspect initial data, individual classifiers for each attack type, and an integral classifier for making a final decision on the presence and attack type in the network traffic. For this, training and testing samples were formed, covering several attacks, namely SYN port scanning, SYN portsweep and SYN flood. A two-step approach was used. In the first step, the sessions were grouped by time intervals and were used for the type of attack recognition. In the second step, the separated sessions of time intervals were categorized as belonging either to attacks or benign activity to enhance the quality of the results. The disadvantage of this approach is the multi-classification error that occurs when a single time interval contains several types of attacks.

The results of the experiment showed that the proposed concept of the multi-aspect approach to detecting and analyzing attacks in IoT clouds allows one to achieve high quality of attack detection and recognition of their type. In addition, the simultaneous use of aspect classifiers based on time periods and separate sessions allows the advantages of these approaches to be combined and their disadvantages levelled.

## 7. Discussion

This article proposes the concept of a multi-aspect approach to detecting and analysis of attacks in IoT clouds. The advantages of this approach are, first, the ability to flexibly change and expand the existing complex detection system by adding extra aspect classifiers to the existing ones; second, the ability to obtain additional information about detected attacks; and third, the improvement of the detection quality indicators due to such expansion and combination of classifiers.

In general, as practice shows, for each specific type of attack often there is an aspect classifier with the best accuracy among the available ones. Therefore, a priori, for a given type of attack, such an aspect classifier is the most preferable to use. For example, among the available aspect classifiers, typical scanning attacks can be most qualitatively detected by using an aspectual host-based classifier of outgoing traffic. First of all, this is due to the fact that the classification of such traffic as attacking or benign traffic is determined not so much by the structure of each session as by the set of outgoing network connections. In contrast, for instance, attacks of exploitation of a certain type of vulnerability, including zero-day vulnerabilities of a certain type of IoT devices, can be detected better per session or by using IoT device type aspects.

However, as it was possible to check with the help of the experiments, the simultaneous consideration of two or more available aspect classifiers allows us to increase the values of the detection quality indicators to some extent, even significantly in some cases. This, therefore, implies the practicability of composing namely multidimensional classifiers for detecting a wide range of attacks in IoT clouds, assuming the formation of means for selecting specific aspects and/or their combination.

To combine various aspect classifiers, a rule-based approach was used in this work. Using the example of several specific types of attack for each of the attacks, several suitable classifiers were identified by expert means. Besides, for these classifiers, the way and sequence of their application for the most accurate revelation of the attacking traffic elements were specified. For instance, the host-based aspect of the incoming traffic made it possible to more accurately detect flooding influences than the session-based aspect. At the same time, in its internal work, this host-based classifier (in fact, the primary classifier) for the flooding attack was oriented mainly on the quantitative features of the incoming traffic of a victim host per unit of time. The session-based aspect classifier (secondary classifier) operates using the structural features of each session separately. In addition to establishing the fact of an attack in a certain period of time, this secondary classifier made it possible to increase the detection accuracy by indicating which particular sessions are attacking and which are not. Thus, the primary classifier allowed us to establish the fact of the presence of a specific attack type within a certain time interval, while the secondary classifier made it possible to clarify which sessions during this interval should be considered as attacks, and which should be regarded as benign actions. Therefore, based on the results of the experiments, we can conclude that for each attack in IoT clouds, for its successful detection it is necessary to select or build, firstly, such an aspect classifier that detects it most efficiently and secondly, one or more aspect classifiers that will improve the precision and recall of its detection.

It should be noted that the novelty of the proposed approach lies mostly in the used feature space. The proposed approach uses different aspects of the input data to enhance the quality of the attacks recognition and to enhance the general attack detection quality.

However, a possible drawback of this approach is regarded as (i) a need for manual analysis of the results of detecting attacks of each type in the tuning process of the detection mechanism, (ii) conducting appropriate experiments, and (iii) forming rules for combining aspect classifiers based on them. To overcome this challenge, it seems appropriate to expand the present approach in the following way. This approach is proposed to be extended by replacing rule-based combining with an ensemble of the aspect classifiers [37,38]. This will also require some experimentation, but nevertheless will allow the combination process to be significantly automated. In comparison with the rule-based one, the advantages of this combined approach can also include obtaining potentially better quality detection indicators. This may firstly be due to a decrease in value of the factor of possible errors in the formation of a system of combination rules; secondly to their obsolescence, i.e., attacks may evolve over time, reducing the effectiveness of specific rules and the numerical values used in them; and finally, reduction of organizational and technical costs for reconfiguring the combining mechanism.

As a result of the above, we present a quality-based comparison of our proposed approach to detecting attacks in the IoT cloud to the relevant alternative approaches and solutions published to date. Table 4 summarizes the main factors on which the comparison is made, alongside specific estimation values. The following range of factors was used, namely the multidimensional nature of the detection mechanism, detection in the cloud, possibility of detection on limited initial data, differentiation of attack types, and flexibility of the detection mechanism.

(1) As the multidimensional nature of the attack detection mechanism, it is expressed in an ability to use various aspects of the initial data to provide multi-classification by using ensembles and selection of the necessary hyperparameters this ability makes it possible to increase the detection quality indicators.
(2) Detection of attacks on the side of the cloud. It determines the possibility of multiple detections of attacks based on parallel monitoring of input devices from various IoT networks working in conjunction with this cloud. In particular, this provides the detection of multistep and coherent attacks, which would be problematic to detect, handling data from separate IoT networks only.
(3) Limited initial data. Due to the privacy of user data and data about specific IoT devices, which for legal reasons should not fall into the cloud, the initial data for the detection mechanisms and the structure of the transmitted data turn out to be significantly restricted. In particular, owing to the imposed business constraints and limitations on the amount of data that can be legally transferred to the cloud, only a limited portion of such raw data can be delivered to the cloud, even after the application of data anonymization.
(4) Differentiation of attack types, i.e., the ability to detect not only an attack itself but also its type.
(5) Flexibility of the detection mechanism, i.e., an ability to quickly configure and rebuild the integrated attack detection mechanism. This quality appears in cases of a change in the nature of the initial data and/or in the need to update or embed knowledge about new types of attack into the detection mechanism. In essence, this flexibility is expressed in adding or changing some aspect classifier into a complex defense mechanism with retraining of only the head classifier without the need to retrain the entire ensemble. In particular, this circumstance greatly simplifies the process of maintaining the attack detection mechanism for operating large-scale IoT networks, where the suspension of security monitoring processes would be critical.

In Table 6, we indicate the feasibility of factors on the basis of the analysis of the respective studies. Five indicated papers used for comparison were selected as the ones most closely related to the topic of this study. In many cases, the information we needed about the decisions was not explicitly stated, and we indicated the value based on the logic of the presentation of the material and the context of the paper. However, in general, the data presented in the papers was sufficient to make this comparison, albeit with some small assumptions—when desirable explicit information was not available or not enough.

Thus, quality-based comparisons of the proposed multi-aspect based approach expose that for each of the five identified factors, this approach is not worse than its analogs. At the same time, when all these factors are taken into account in total, our approach is superior to the modern analogs considered. Moreover, the testing made it possible to establish that, on average, in conditions of significant limitation of the initial data delivered to the cloud, due to its privacy, nevertheless, our final classifier shows values of the detection quality comparable to analogs.

## 8. Conclusions

This article solves the problem of effective attack detection in IoT clouds on the basis of the analysis of their traffic by the means of cloud infrastructure. The training models built are based on a combination of multi-aspect classifiers, which made it possible to improve the detection quality indicators. Each ensemble of multi-aspect classifiers is a meta-algorithm consisting of all the individual machine learning methods used. The way they are used together can be different (voting, boosting, stacking, etc.) and their usage is chosen to ensure the efficiency of the final solution. This approach allows us to preserve all the advantages of individual methods and neutralize their disadvantages.

Summarizing the proposed approach, it in general has two advantages (it can use the statistical information about session instead of the full traffic and this allow one to partially solve the data privacy issues and reduce the amount of the transmitted data) and one disadvantage—the incompleteness of the available in-the-cloud data can reduce the quality of attack detection. The main contribution of the proposed approach is the ability to recognize not only the fact of the attack but also to get additional information regarding the attack type.

The experimentally obtained improvements of the indicators of precision, recall, and f1-measure for each type of attack in question suggest the potential prospects of the underlying approaches for constructing tools of detecting volumetric and multistep attacks on IoT clouds, including botnets and attacks using zero-day vulnerabilities.

In the future, we are planning to extend the list of the constructed features based on various aspects of network traffic. We believe this will allow us to enhance the quality of attack type detection. We are also planning to extend the list of attacks in experiments to evaluate the detection quality in various cases.

## Figures and Tables

**Table 1:** Traffic features for attack detection.

No	Feature	Description
1	actualTime	Reporting time
2	Gateway MAC	Gateway MAC
3	MAC	Connected device MAC
4	connectPort	Connection port
5	srcIP	NAT LAN source address
6	srcPort	NAT LAN source port
7	Protocol	Protocol TCP/UDP
8	destIP	Destination address after NAT mapping
9	destPort	Destination port after NAT mapping
10	flag	The flag of the protocol header, which takes the AND value of A|F|U|P|R|S|C|E
11	status	Session state
12	inBytes	Upstream incremental bytes
13	outBytes	Downstream incremental bytes
14	inPkts	Number of upstream incremental packets
15	outPkts	Number of downstream incremental packets
16	MIN_IP_PACKET_TOTAL_LENGTH_UP	Minimum upstream IP packet length
17	MIN_IP_PACKET_TOTAL_LENGTH_DWN	Minimum downstream IP packet length
18	MAX_IP_PACKET_TOTAL_LENGTH_UP	Maximum upstream IP packet length
19	MAX_IP_PACKET_TOTAL_LENGTH_DWN	Maximum downstream IP packet length
20	octetVarianceUp	Upstream bytes variance statistics
21	octetVarianceDwn	Downstream byte variance statistics
22	flowTimeIntervalUp	Upstream average interval time
23	flowTimeIntervalDwn	Downstream average interval time
24	FLOW_TIME_VARIANCE_UP	Intra-stream uplink interval variance
25	FLOW_TIME_VARIANCE_DWN	Intra-Stream Downstream Interval Variance
26	fragmentPacketCountUp	Number of upstream IP fragmented packets
27	fragmentPacketCountDwn	Number of downstream IP fragment packets
28	MIN_TTL_UP	Minimum upstream TTL
29	MIN_TTL_DWN	Downlink bottom TTL
30	MAX_TTL_UP	Maximum upstream TTL
31	MAX_TLL_DWN	Maximum downstream TTL
32	TCP_OUT_OF_ORDER_BYTES_UP	TCP upstream out-of-order statistics
33	TCP_OUT_OF_ORDER_BYTES_DWN	TCP out-of-order statistics

**Table 2:** Traffic features for detection of DDoS attacks.

No	Feature	Description
1	startTime	Reporting time.
2	destIP	Destination IP address
3	destPortAmount	Amount of different destination ports
4	srcIPAmount	Amount of different source IP addresses
5	srcPortAmount	Amount of different source ports
6	sessions1Amount	Amount of sessions with 1 packet
7	sessionsWODataAmount	Amount of sessions without packets that don’t have any TCP flags
8	sessionsAmount	Total amount of sessions
9	closedSessionsAmount	Amount of successfully closed sessions
10	halfOpenedSessionsAmount	Amount of halfOpened sessions
11	timeoutedSessionsAmount	Amount of sessions closed by timeout
12	inBytes	Upstream incremental bytes
13	outBytes	Downstream incremental bytes
14	inPkts	Number of upstream incremental packets
15	outPkts	Number of downstream incremental packets

**Table 3:** Classification quality for 1_portsweep_warezclient.csv.

Name of Method	isAttack	Precision	Recall	f1-Score	1-FPR = TNR
AdaBoostClassifier	0	0.92	1.00	0.96	1.000
	1	0.98	0.16	0.28	
RandomForestClassifier	0	0.92	1.00	0.96	1.000
	1	0.98	0.16	0.28	
MultinomialNB	0	0.94	0.26	0.40	0.255
	1	0.10	0.84	0.18	
LogisticRegression	0	0.96	0.26	0.40	0.256
	1	0.11	0.88	0.19	
SVM	0	0.93	0.20	0.33	0.203
	1	0.09	0.84	0.17	
DecisionTreeClassifier	0	0.92	1.00	0.96	0.999
	1	0.94	0.16	0.28	
RidgeClassifier	0	0.91	1.00	0.95	1.000
	1	0.00	0.00	0.00	

**Table 4:** Hyper-parameters of the RandomForestClassifier algorithm.

Name of Parameter	Value
criterion	entropy
max_depth	8
min_samples_leaf	1
n_estimators	50

**Table 5:** Analysis of the correlation between features.

Feature Name	Feature Importance
SYN	0.296271
ACK	0.273176
Status(NormalSIN,NormalFIN,RST)	0.077425
Src_MIN_LENGTH	0.069216
NormalRST	0.06094
Src_MAX_LENGTH	0.041913
Src_LENGTH	0.030735
Dst_MIN_LENGTH	0.030077
Dst_MIN_TLL	0.023558
Dst_MAX_LENGTH	0.016709
Dst_MAX_TTL	0.016132
NormalSYN	0.014827
RST	0.013032

**Table 6:** Comparison with relevant approaches and solutions.

Factor	Kanev 2017 [27]	Larriva-Novo, 2020 [21]	Sicato 2020 [32]	Branitsky 2020 [37]	Kiran 2020 [29]	This Approach
Multi-aspect nature of the detection mechanism	+	+	-	+	-	+
Detection in the cloud	-	-	+	-	+	+
Limited initial data	-	-	-	-	-	+
Differentiation of attack types	-	+	+	+	-	+
Detection mechanism flexibility	+	+	-	-	-	+

## Data Availability

Not applicable.

## Abbreviations

The appendix represents the following full forms for the abbreviations used in the article.

IoT	Internet of Things
GUI	Graphical User Interface
TCP	Transmission Control Protocol
IP	Internet Protocol
SSH	Secure Shell
HTTP	HyperText Transfer Protocol
MAC	Media Access Control
NAT	Network Address Translation
LAN	Local Area Network
UDP	User Datagram Protocol
TTL	Time to Live
ROC	Receiver Operating Characteristic
DDoS	Denial of Service
PCAP	Packet Capture
FPR	False Positive Rate
TNR	True Negative Rate
SVM	Support Vector Machine
DARPA	Defense Advanced Research Projects Agency
SYN	Synchronization TCP flag
